# The Adaptor Protein Alix is Involved in the Interaction Between the Ubiquitin Ligase NEDD4-1 and its Targets, ABCG1 and ABCG4

**DOI:** 10.3390/ijms20112714

**Published:** 2019-06-02

**Authors:** Amjad Alrosan, Shereen M. Aleidi, Alryel Yang, Andrew J. Brown, Ingrid C. Gelissen

**Affiliations:** 1 Sydney Pharmacy School, Faculty of Medicine and Health, The University of Sydney, Sydney, NSW 2006, Australia; aalr3235@uni.sydney.edu.au (A.A.); zyan8789@uni.sydney.edu.au (A.Y.); 2 School of Pharmacy, The University of Jordan, Amman 11942, Jordan; S.Aleidi@ju.edu.jo; 3 School of Biotechnology and Biomolecular Sciences, The University of New South Wales, Sydney, NSW 2052, Australia; aj.brown@unsw.edu.au

**Keywords:** ABCG1, ABCG4, NEDD4-1, Alix, cholesterol homeostasis

## Abstract

Several ATP-Binding Cassette (ABC) transporters, including ABCG1 and the related ABCG4, are essential regulators of cellular lipid homeostasis. ABCG1 is expressed ubiquitously and is functional in the context of atherosclerosis. However, ABCG4 is expressed almost exclusively in brain and has been linked to Alzheimer’s disease (AD). These transporters are highly regulated post-translationally by E3 ubiquitin ligases, with the ligase NEDD4-1 (Neural precursor cell-expressed developmentally downregulated gene 4) implicated in their protein stability. In this study, we investigated interacting partners of ABCG1 using peptide-mass spectrometry and identified the potential adaptor protein, Alix (apoptosis-linked gene 2-interacting protein X). In this paper, we hypothesized and investigated whether Alix could facilitate the interaction between NEDD4-1 and the ABC transporters. We showed that Alix and NEDD4-1 proteins were co-expressed in several commonly used cell lines. Knockdown of Alix in cells overexpressing ABCG1 or ABCG4 increased transporter protein expression while co-immunoprecipitation experiments showed interaction between NEDD4-1, Alix, and ABC transporters. In summary, we provide evidence that Alix serves as a co-factor for the interaction between the E3-ubiquitin ligase NEDD4-1 and the ABC transporter targets, ABCG1 and ABCG4.

## 1. Introduction

Cholesterol is essential for many cellular processes, including the production of cell membranes and synthesis of hormones. However, an imbalance in cellular cholesterol homeostasis increases the risk of a number of chronic conditions, such as atherosclerosis and Alzheimer’s disease (AD) [1,2]. In response to high cholesterol levels, cells can upregulate their expression of cholesterol export proteins, which are members of the ATP binding cassette subfamily, or ABC transporters, including ABCA1, ABCG1, and its close relative ABCG4 [3,4].

ABCA1 and ABCG1 are expressed ubiquitously while ABCG4 is expressed mostly in astrocytes and neurons in the brain [5]. ABCA1 and ABCG1 have been linked to atherosclerosis, and their potential substrates besides cholesterol include phospholipids as well as oxysterols [5,6]. ABCG4, which has been thought to export cholesterol, oxysterols, and cholesterol synthesis intermediates, has been linked to AD due to its additional proposed role in amyloid-β peptide export from brain cells [7]. These transporters donate their cargo to extracellular acceptors. ABCG1 is thought to transport cholesterol to high density lipoprotein (HDL) while ABCA1 mediates transport of cholesterol and phospholipids to lipid-poor HDL (mostly apolipoprotein A1) in circulation [8,9,10]. In the brain, ABCA1 and ABCG1 concurrently enhance cholesterol efflux from neurons to apolipoprotein E, a process that is thought to reduce the synthesis of Aβ peptides [7].

It has been suggested that these transporters are highly regulated at the post-translational level. One of the post-translational mechanisms involved is protein ubiquitination, facilitated by E3 ubiquitin ligases, which specifically target proteins for proteasomal degradation [11,12,13]. Previous work from our laboratory has identified and investigated the roles of three E3 ligases belonging to the Homologous to E6-AP Carboxyl Terminus (HECT)-domain subfamily (namely HECTD1, HUWE1, and Neural Precursor Cell-expressed Developmentally Downregulated Gene 4 or NEDD4-1) in the regulation of ABC lipid transporter activity and stability [14,15]. Considering that these E3 ligases already have important functions in the regulation of other proteins, any mechanistical understanding of how they interact with ABC transporters could provide a more specific approach to future targeting strategies. The substrate specificity of HECT-domain E3 ligases is determined by specific domains located in the N-terminal region of the E3 ligase [12,13]. NEDD4-1 is the prototypical member of the NEDD4 subfamily, which is the largest subfamily of HECT-domain E3s [12,16]. Human NEDD4-1 contains an N-terminal C2 domain (calcium dependent phospholipids binding domain) and four tryptophan-tryptophan (WW) domains, which are important in the interaction with the target protein substrates [12,13,17]. The mechanism by which HECT-domain E3 ligases interact with their substrates is poorly understood. However, some target protein substrates contain consensus sequences or motifs, which are rich in proline amino acids and can interact with the WW domain of the E3 ligase. These consensus sequences include PPxY (Proline-Proline-x-Tyrosine, where “x” is any amino acid) domains [18], which are the most common [19].

In order to understand how the E3 ligases that we identified might interact with ABCG1 and ABCG4, we examined whether ABCG1 and/or ABCG4 have proline-rich consensus sequences, and found that these were absent. Therefore, we hypothesized that potential adaptor proteins containing proline-rich consensus sequences might mediate the interaction between the ligases that we identified and our ABC lipid transporter targets. In this follow-up study, we identified one potential adaptor protein, Alix (apoptosis-linked gene 2-interacting protein X), from a mass-spectrometry screen of proteins interacting with ABCG1. Alix was previously shown to be capable of serving as an adaptor between NEDD4-1 and another target protein [20], so we subsequently investigated whether Alix could facilitate the interaction between ABCG1, ABCG4 and NEDD4-1.

## 2. Results

### 2.1. Identification of Alix in a Mass Spectrometry Screen

We have previously described the identification of three E3-ubiquitin ligases as interacting partners with ABCG1 [14,15]. Briefly, ABCG1-CHOK1 cells were harvested and ABCG1 protein was subjected to immunoprecipitation (IP), with the IP products digested and analyzed for interacting proteins via peptide mass spectrometry [14]. We re-evaluated the mass spectrometry results (the methodology for which is described in detail in [14]), including a number of peptide hits that were previously excluded on the basis of our stringent selection criteria (Mascot score of >30 with a minimum of 2 independent peptide hits [14]). We specifically looked for proteins that were previously identified as known adaptor proteins for NEDD4 family members, and amongst the candidates we found the protein adaptor Alix (or Programmed cell dead 6-interacting protein, AIP1 or ALG-2-interacting protein). This candidate received a Mascot score of 39, with 2 independent peptide hits. One of the peptide hits, which was a small fragment, was also present in another unrelated protein, hence this candidate was not listed in the Supplementary Table in our original publication [14]. Considering that Alix was previously identified as an adaptor for NEDD4-1 [20], we decided to investigate this protein to see whether it could serve as a co-factor for ABCG1 as well as ABCG4.

### 2.2. Expression of Alix and NEDD4-1 in Various Cell Lines

**Figure 1:**
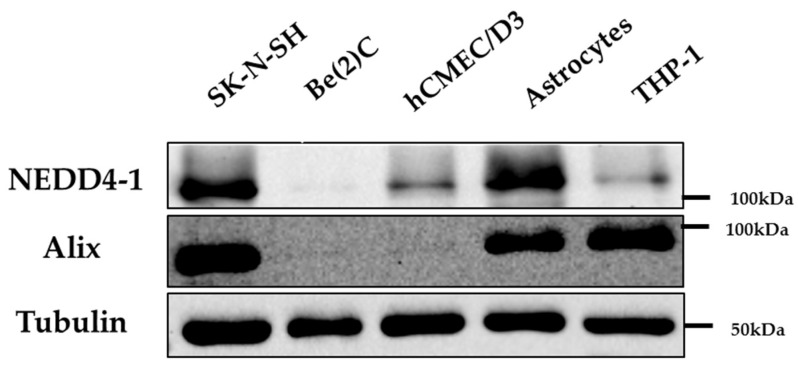
Expression of Apoptosis-linked gene 2-interacting protein X (Alix) and Neural precursor cell-expressed developmentally downregulated gene 4 (NEDD4-1) protein in various cell lines. Immunoblots of Alix and NEDD4-1 protein expression in cell lines as listed in Materials and Methods (6 µg cell protein loaded per lane).

Alix has been shown to be widely expressed in many cell types [21,22]. Our first aim was to confirm whether both Alix and NEDD4-1 were co-expressed in cell lines of various origins, including brain cell lines and THP-1 macrophages where ABCG1 is highly expressed. Firstly, of the two cell lines that were of neuronal origin, only SK-N-SH had high expression of both Alix and NEDD4-1, while Be(2)C had very little expression of either proteins in comparison (Figure 1). Astrocytes also expressed both proteins, however, both were poorly expressed in hCMEC/D3 cells. Lastly, THP-1 macrophages expressed comparable levels of Alix but low levels of NEDD4-1, indicating that there are cell-specific differences in expression levels.

### 2.3. SiRNA Knockdown of Alix Increases ABCG1 Protein Expression

**Figure 2:**
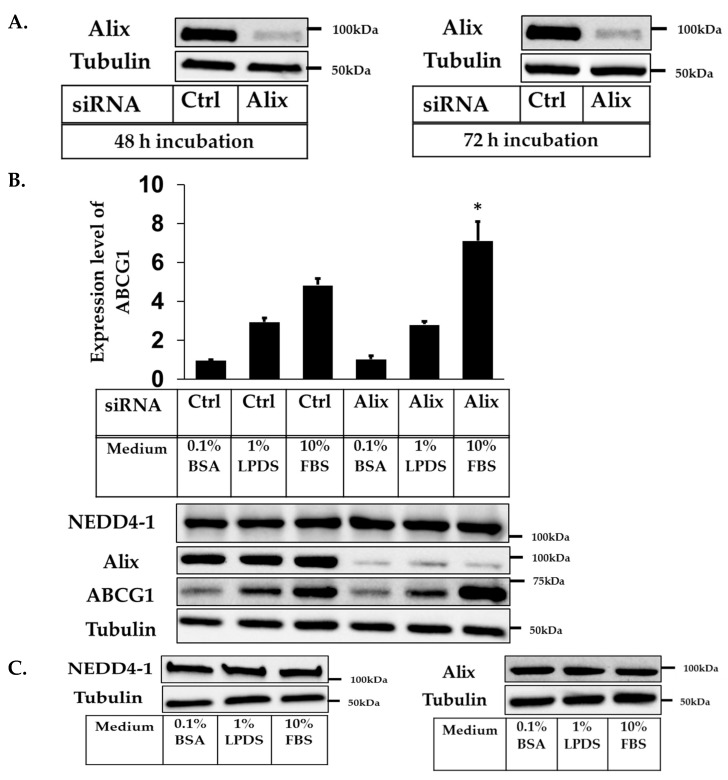
siRNA knockdown of Apoptosis-linked gene 2-interacting protein X (Alix) increases ABCG1 protein expression. (**A**) Immunoblots of cell lysates from ABCG1-CHOK1, transfected with either control or Alix siRNA. (**B**) Quantification of ABCG1 protein relative to tubulin (expressed as fold change relative to control 0.1% BSA) in low and normal serum conditions (mean ± SEM of seven data points from four independent experiments). * indicates *p* < 0.05 compared to control in 10% FBS. (**C**) Immunoblots of Alix and Neural precursor cell-expressed developmentally downregulated gene 4 (NEDD4-1) from cell lysates of ABCG1-CHOK1 in low and normal serum conditions.

As mentioned, we have previously shown that knockdown of NEDD4-1 in ABCG1-CHOK1 cells increased ABCG1 protein expression and activity. To examine the role of Alix in this interaction between ligase and target, we set up siRNA-mediated knockdown of Alix in these cells. Optimization of transfection conditions was performed and showed that cells harvested 48 or 72 h post-transfection resulted in equivalent efficiency of Alix knockdown (Figure 2A). Hence, we used 48 h as post-transfection time for all further experiments utilizing ABCG1-CHOK1.

Next, we measured the consequence of a reduction in Alix protein levels on ABCG1 expression. Considering that we previously showed that ABCG1 protein stability is dependent on the cell cholesterol status [23], these experiments were performed under three different conditions that varied cellular cholesterol content. As expected, ABCG1 protein levels were highest in cells grown in full serum and lowest in cells grown in 0.1% BSA (Figure 2B). After Alix knockdown, cells grown in normal serum containing growth media (i.e., 10% FBS) displayed significantly increased ABCG1 protein levels (approximate 1.5-fold) compared to cells transfected with control primers. However, this effect was not observed when cells were cultured overnight in serum-deprived conditions i.e., either 1% LPDS or 0.1% BSA (Figure 2B). To exclude the possibility that these changed media conditions affected baseline Alix levels, we measured Alix as well as NEDD4-1 basal levels in untransfected cells and showed that these were unchanged for all three different media conditions (Figure 2C). In summary, these results show that knockdown of Alix in ABCG1-CHOK1 significantly increased ABCG1 protein expression, but only when cells were maintained in normal serum-containing media. These effects were not seen when cells were deprived of cholesterol and other lipids present in normal serum-containing media.

### 2.4. SiRNA Knockdown of Alix Increases ABCG1 Protein Expression after Cholesterol Loading

**Figure 3:**
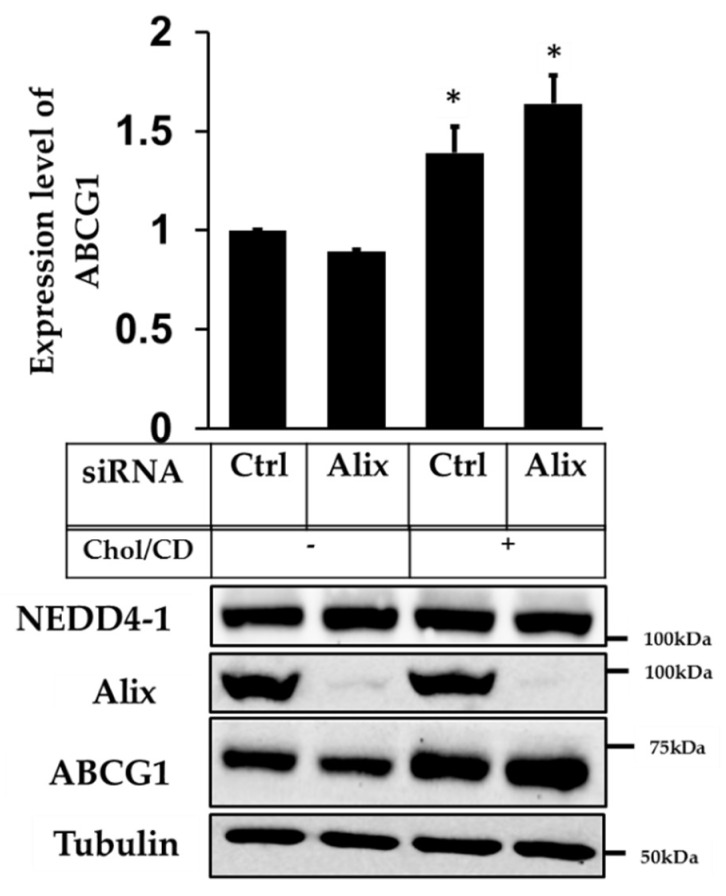
Effect of Apoptosis-linked gene 2-interacting protein X (Alix) knockdown on ABCG1 protein expression in ABCG1-CHOK1, with and without cholesterol loading. Representative immunoblot of ABCG1-CHOK1, transfected with either control or Alix siRNA, and treated for 6 h with or without Chol/CD (20 μg/mL) in 0.1% BSA. Mean ± SEM of 3 individual data points from two independent experiments, expressed as fold change relative to control without chol/CD. * indicates *p* < 0.05 compared to control.

Considering the previous findings that Alix knockdown only increased ABCG1 levels when performed in serum (which is a complex mixture of nutrients and lipids, including cholesterol), we investigated next whether cholesterol addition itself would be sufficient to stabilize ABCG1 levels under the conditions tested. To examine this, cells were incubated with control or Alix primers for 48 h and then loaded with or without 20 µg/mL of cholesterol/cyclodextrin (Chol/CD) complexes in 0.1% BSA background for 6 h. Figure 3 shows that addition of cholesterol alone for 6 h increased ABCG1 protein expression in the control condition, as expected from our previous published work [23]. In cells where Alix expression was reduced, this effect was significantly enhanced even further, suggesting that Alix is associated with a cholesterol-sensitive ABCG1 protein pool.

### 2.5. Alix and NEDD4-1 Co-Immunoprecipitate with ABCG1

**Figure 4:**
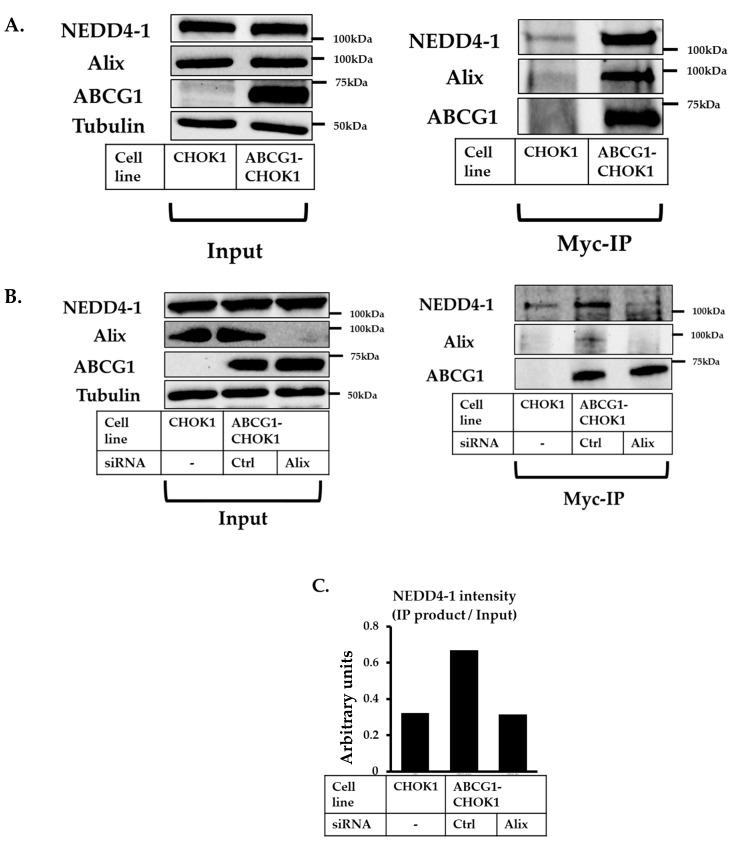
Apoptosis-linked gene 2-interacting protein X (Alix), Neural precursor cell-expressed developmentally downregulated gene 4 (NEDD4-1) and ABCG1 co-immunoprecipitation (co-IP). (**A**) IP of CHOK1 and ABCG1-CHOK1 cell lysates, immunoprecipitated with anti-myc antibody. Results are representative of two independent experiments. (**B**) IP of CHOK1 and ABCG1-CHOK1 cell lysates after knockdown of Alix (48 h), immunoprecipitated with anti-myc antibody. Results are representative of two independent experiments. (**C**) represents quantification of NEDD4-1 in IP product presented in Figure B, with/without Alix knockdown, relative to NEDD4-1 in whole cell lysate (Input).

To investigate whether Alix could potentially serve as an adaptor protein, facilitating the interaction between ABCG1 and NEDD4-1, we used a co-immunoprecipitation (co-IP) approach to look for evidence of an Alix, ABCG1, and NEDD4-1 complex (Figure 4). Immunoblots of cell lysates showed that Alix and NEDD4-1 were present both in parent CHOK1 cells and ABCG1-CHOK1 while ABCG1 was absent from CHOK1 cell lysates (Figure 4A, left panel; input). IP products showed the presence of Alix as well as NEDD4-1 protein, which were present in significantly reduced amounts in CHOK1 cells (Figure 4A, right panel; IP), despite being present in equal amounts in starting cell lysates (left panel, input). After Alix knockdown, significantly less NEDD4-1 was found to be co-migrating with ABCG1 (Figure 4B and quantified in Figure 4C). Taken together, these data provide evidence that Alix is present in a complex with ABCG1 and NEDD4-1, hence is a likely co-factor for NEDD4-1.

### 2.6. Knockdown of Alix Decreases ABCA1 Protein Expression

**Figure 5:**
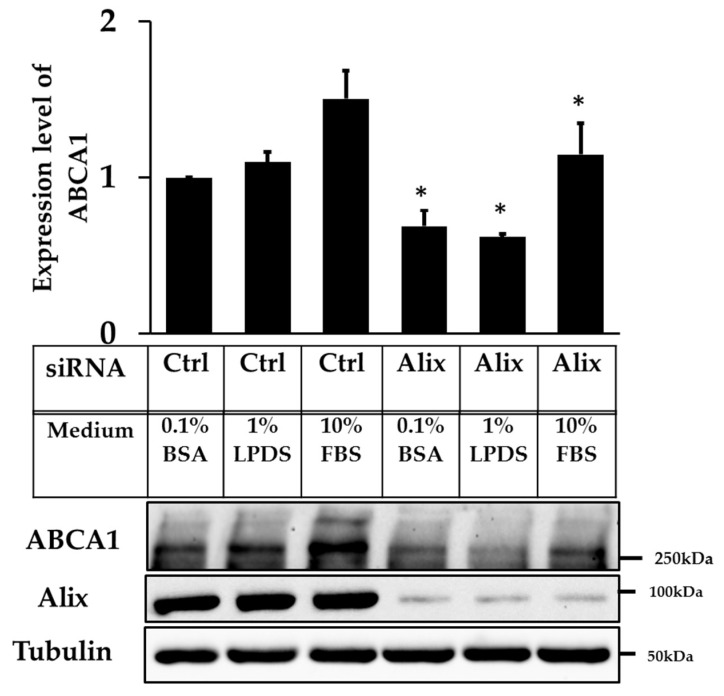
siRNA knockdown of Apoptosis-linked gene 2-interacting protein X (Alix) decreases ABCA1 protein expression. Quantification of ABCA1 protein relative to tubulin in low and normal serum conditions in ABCG1-CHOK1 (mean ± SEM of 4 individual data points from three independent experiments, expressed as fold change compared to control 0.1% BSA). * indicates *p* < 0.05 compared to control.

As for ABCG1, ABCA1 protein stability can be affected by cellular cholesterol levels [23], hence we examined whether Alix knockdown and the subsequent increase in (overexpressed) ABCG1 protein and activity affected endogenous ABCA1 protein expression. ABCG1-CHOK1 cells were incubated with control or Alix siRNA for 48 h and incubated overnight with low serum conditions (i.e. 1% LPDS and 0.1% BSA) before harvesting. Endogenous ABCA1 protein level was decreased to ~50-60% of control levels in low and normal serum media (Figure 5), indicating that there was compensatory drop in ABCA1 levels due to the reduction in cholesterol status of the cells.

### 2.7. SiRNA Knockdown of Alix Increases ABCG4 Protein Expression

**Figure 6:**
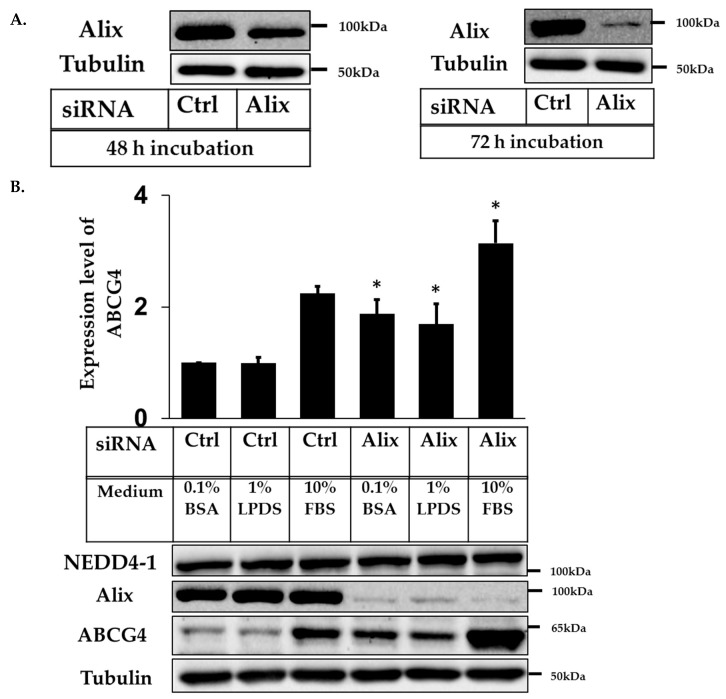
siRNA knockdown of Apoptosis-linked gene 2-interacting protein X (Alix) increases ABCG4 protein expression. (**A**) Immunoblots of cell lysates from ABCG4-CHOK1, transfected with either control or Alix siRNA. (**B**) Quantification of ABCG4 protein relative to tubulin in low and normal serum conditions (mean ± SEM of three independent experiments, each performed in duplicate cultures, and expressed as fold change relative to control 0.1% BSA). * indicates *p* < 0.05 compared to control.

Similar to ABCG1, NEDD4-1 was previously shown to be involved in the post-translational processing of ABCG4 [14]. Our next aim was to investigate whether Alix was also relevant in the interaction between ABCG4 and NEDD4-1. Since the half-life of ABCG4 was found to be longer than ABCG1 [14], the transfection conditions were optimized first as for ABCG1. The optimal time for incubation of cells with Alix primers was found to be longer for ABCG4-CHOK1 cells, with 72 h of transfection required to get adequate stabilization of ABCG4 levels (Figure 6A). Hence, we used 72 h as post-transfection time for all next further experiments in ABCG4-CHOK1.

Surprisingly, although ABCG1 protein expression was increased significantly after Alix knockdown only in 10% FBS, ABCG4-CHOK1 grown in full and low serum conditions showed significantly increased ABCG4 protein levels of approximate 1.5-fold after Alix knockdown (Figure 6B). These results show that knockdown of Alix in ABCG4-CHOK1 significantly increased ABCG4 protein expression, when cells were maintained in normal and low serum-containing media.

### 2.8. Alix and NEDD4-1 Co-Immunoprecipitate with ABCG4

**Figure 7:**
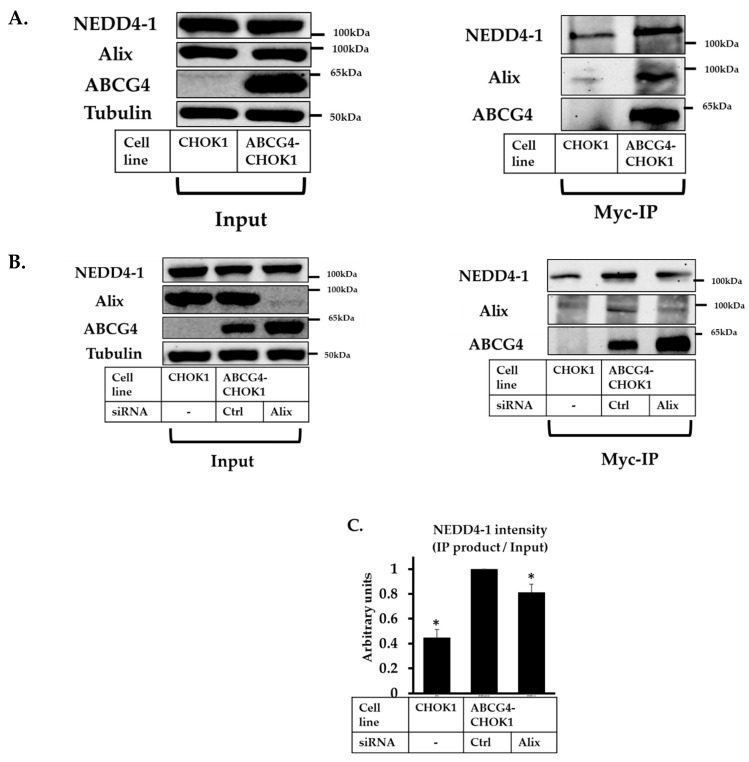
Apoptosis-linked gene 2-interacting protein X (Alix), Neural precursor cell-expressed developmentally downregulated gene 4 (NEDD4-1) and ABCG4 co-immunoprecipitation (co-IP). (**A**) IP of CHOK1 and ABCG4-CHOK1 cell lysates, immunoprecipitated with anti-myc antibody. Results are representative of two independent experiments (**B**) IP of CHOK1 and ABCG4-CHOK1 cell lysates after knockdown of Alix (72 h), immunoprecipitated with anti-myc antibody (**C**) represents quantification of NEDD4-1 in IP product, with/without Alix knockdown, relative to NEDD4-1 in whole cell lysate (Input) from three independent data points (* indicates *p* < 0.05 relative to the ABCG4-CHOK1 control transfected cells).

As described for ABCG1, we used an IP approach to investigate if Alix may form a complex with NEDD4-1 and ABCG4. Western blots of pre-IP cell lysates showed the presence of Alix and NEDD4-1 protein in both parent CHOK1 and ABCG4-CHOK1, but ABCG4 was absent from CHOK1 cell lysates (Figure 7A, left panel; input). Immunoprecipitating myc-tagged ABCG4 from ABCG4-CHOK1 cells indicated enrichment of NEDD4-1 and Alix (Figure 7A, right panel; IP). No Alix was observed in the control IP of CHOK1 cells, and a significant reduction in the level of NEDD4-1 in CHOK1 parental cells compared to ABCG4-CHOK1. Although the findings were not as striking as for ABCG1, Alix knockdown did decrease NEDD4-1 levels co-immunoprecipitating with ABCG4, suggesting that Alix may also facilitate the interaction between ABCG4 and NEDD4-1 (Figure 7B and quantification in Figure 7C).

## 3. Discussion

In the current study, we characterized the role of the protein Alix as an adaptor protein that can mediate the interaction between NEDD4-1 and two ABC lipid transporters, ABCG1 and ABCG4. Alix is a cytoplasmic protein that plays a key role in endo-lysosomal trafficking through interaction with proteins of the endosomal sorting complexes required for transport (ESCRT) machinery [24]. Previously, Alix was shown to act as an adaptor protein recruiting NEDD4-1 to the vicinity of HIV-1 Gag protein, which is a NEDD4-1 substrate that lacks a PPxY motif [20]. Sette et al [20] demonstrated that NEDD4-1 enhancement of HIV-1 release required Alix, which binds to the HIV-1 Gag protein [20]. In addition, they showed that NEDD4-1 binds and ubiquitinates Alix in the cell, suggesting a NEDD4-1–Alix physical and functional interdependence [20]. These findings support a model in which Alix recruits NEDD4-1 to facilitate the budding of the HIV-1 virus from cells [20].

Alix has previously been shown to contain a C-terminal proline-rich sequence domain (PPxY), through which it can bind with targets such as ALG-2 (apoptosis-linked gene 2), involved in inducing neuronal death. Deletion of the proline-rich motif was shown to block binding and thus protects neurons from apoptosis [24,25]. It has furthermore been suggested that Alix has a central role in the normal development of the mouse brain [26].

So far, only one report has implicated a role for Alix in cholesterol homeostasis, with an indirect role for Alix being described in maintenance of cholesterol level in the late endosomal compartment through its binding with lysobisphosphatidic acid (LBPA) [27]. Here, we add further evidence that Alix has a role to play in cholesterol homeostasis by facilitating the interaction between NEDD4-1 and ABCG1/ABCG4. We found that Alix and NEDD4-1 were co-expressed in a number of brain cell lines, including neuronal cells and astrocytes. One of the limitations of this work is that we were unable to measure the interaction of Alix with the endogenous ABCG1 and ABCG4 proteins, due to the lack of commercial antibodies with the required sensitivity. Using an overexpression model (i.e., no transcriptional compensation of ABCG1 or ABCG4 expression), we confirmed that Alix knockdown led to increased protein levels and activity of a cholesterol-regulated ABCG1 protein pool. In addition, we confirmed with a co-IP approach that all three proteins were interacting, and that there was a loss of ABCG1/NEDD4-1 interaction when Alix levels were depleted. Similar results were found for ABCG4, a transporter that has been indicated in regulating sterol levels in the brain and has been implicated in Alzheimer’s disease as an exporter of amyloid-β peptides from cells [7,28]. Intriguingly, Alix has independently been identified as a possible plasma marker for AD as its level was significantly reduced by around 50% in the cortex and 60% in the hippocampus in an Alzheimer’s mouse model as well as plasma from patients [29]. It is unclear why Alix levels would be depleted and this warrants further investigation.

In summary, we have identified and characterized a novel player in the post-translational processing of ABCG1 and ABCG4, with Alix as a potential cofactor between NEDD4-1 and the ABC transporters. Further experiments are needed to investigate these findings in cells expressing the native transporters. Since these pathways are becoming of more interest for therapeutic targeting, finding specific interactions may facilitate the specificity of approaches to upregulate ABC transporter activity with future drug development in mind.

## 4. Materials and Methods

### 4.1. Materials

Ham’s F-12, Ham’s F12/MEM, MEM, low glucose/DMEM, and RPMI-1640 cell culture media were all purchased from Thermo Fisher Scientific, except EGM2MV medium that was purchased from Lonza Australia (Mount Waverly, Vic, Australia).

Fetal bovine serum (FBS), L-glutamine (200 mM), penicillin (10,000 units/mL), streptomycin (10,000 μg/mL), phosphate buffered saline (PBS), zeocin antibiotic, ethylenediaminetetraacetic acid (EDTA), 0.5% trypsin/ EDTA, bicinchoninic acid (BCA) reagent A, lipofectamine^®^ RNAiMAX, pcDNA3.1myc/his, Dynabeads protein G magnetic beads, and Opti-MEM^®^ I reduced serum media were all purchased from Life Technologies Australia (Mulgrave, Vic, Australia).

Small interfering RNA (siRNA), protease and phosphatase inhibitor cocktails, Bovine serum albumin (BSA) (essentially fatty acid free), bromophenol blue, IGEPAL, cholesterol, copper (II) sulfate pentahydrate, ponceau S stain, ammonium persulfate (APS), and siRNA were all purchased from Sigma-Aldrich (Castle Hill, NSW, Australia). Dithiothreitol (DTT) was purchased from Astral Scientific (Taren Point, NSW, Australia).

Reagents for casting SDS-PAGE gels, including acrylamide, Tris-HCL, glycine, sodium dodecyl sulfate (SDS), and tetramethylethylenediamine (TEMED) were purchased from VWR Life Science (Tingalpa, QLD, Australia). Enhanced chemiluminescent (ECL) reagents, nitrocellulose membrane and hyperfilm ECL were purchased from Millipore, GE healthcare Bio-science, and Amersham. Developer and fixer were purchased from AGFA (Sydney, NSW, Australia).

Anti-ABCA1 monoclonal antibody was from Millipore. Anti-tubulin monoclonal, anti-Alix polyclonal, anti-ABCG4 polyclonal, and secondary anti-mouse and anti-rabbit antibodies were from Sigma-Aldrich. Anti-myc polyclonal was from Abcam. Lipoprotein-deficient serum (LPDS) was generated from FBS as described in Luu et al. [30]. 

### 4.2. Cell Culture

Cell lines were purchased from ATCC (Manassas, VA, USA), Sigma-Aldrich (Castle Hill, NSW, Australia) and CellBank Australia (Westmead, NSW, Australia). Chinese Hamster Ovary (CHOK1) parental and CHOK1 stably overexpressing human myc-tagged ABCG1 (+12) (ABCG1-CHOK1) were described previously [14,31,32]. Human myc-tagged ABCG4 cells were generated as described for human myc-tagged ABCG1 cells. Briefly, cDNA expressing untagged ABCG4, as described in [32] was subcloned into pcDNA3.1 myc/his (Life Technologies Australia, Mulgrave, Vic, Australia), utilizing primers that removed the c-terminal stop codon. CHOK1 parental cells were transfected with ABCG4-cMyc cDNA as described in Gelissen et al. [32] and stable expressors generated as described [14,31,32]. Cells were maintained in Ham’s F12 medium containing 10% (*v*/*v*) heat-inactivated FBS and supplemented with L-glutamine (2 mM), penicillin (100 U/mL), streptomycin (100 µg/mL), and zeocin (200 µg/mL, ABCG1 and ABCG4 overexpressors only) at 37 °C in 5% CO_2_.

Human THP-1 monocytes were maintained in RPMI-1640 medium containing heat-inactivated FBS, L-glutamine, penicillin, and streptomycin with the same concentrations as CHOK1 cells at 37 °C in 5% CO_2_. The neuronal cell line Be(2)C was cultured in Ham’s F12/MEM (50:50, *v*/*v*) while SK-N-SH neurons were grown in low glucose/DMEM plus additions as CHOK1 cells. U87MG microglial cells (referred to further as astrocytes) were maintained in MEM medium plus additions as CHOK1 cells. The blood brain barrier endothelial cell line hCMEC/D3 was maintained in EGM2MV medium plus supplements, which are 5% FBS, hydrocortisone, ascorbic acid, and growth factors as described by the supplier (Lonza Australia (Mount Waverly, Vic, Australia).

### 4.3. siRNA Transfection

CHOK1 cells (either parental or overexpressors) were seeded in 12-well plates one day before transfection in order to achieve 30–40% confluency. Cells were transfected with 0.125 µM control (scrambled) or mouse Alix siRNA using Lipofectamine RNAiMAX at a ratio of 1 µL siRNA per 3 µL lipofectamine in antibiotic free transfection medium. After 24 h, cells were incubated with normal media (10% FBS) containing antibiotics for a further 24 h for ABCG1-CHOK1 or 48 h for ABCG4-CHOK1. In some experiments, cells were incubated with 0.1% BSA and 1% LPDS overnight before harvesting, as described in Figure legends.

### 4.4. Co-Immunoprecipitation (Co-IP)

CHOK1, ABCG1-CHOK1 or ABCG4-CHOK1 were seeded in T75 flasks for 24 h to achieve 80-90% confluency before harvesting. Cells were washed twice with ice cold PBS and lysed in 2 mL of ice cold RIPA buffer [14], containing protease and phosphatase inhibitors cocktails (5 μL/mL). After harvesting and collecting of cell lysates, unbroken cells and cell debris were removed by centrifugation (5 min, 160× *g*). 50 μL of pre-washed magnetic protein G beads were added into 1.5 mL tubes, together with 3 μL of anti-myc antibody in 500 μL of RIPA buffer and incubated on the rotating wheel at 4 °C for 2 h. After 2 h, cell lysates were added (1 mL maximum volume), followed by further overnight incubation on a rotating wheel at 4 °C. The next day, the supernatants were separated from the beads using a magnet. The beads containing the IP products were then washed three times with 1 mL of cold RIPA; the first wash was for one hour, the second wash was for 30 min, and the third wash was for 15 min, followed by one wash with PBS alone to remove excess detergent. Between washes, the tubes were put on the rotating wheel in the cold room at 4 °C. After removing the washes, the IP products were removed from the beads by mixing with 1× SDS-PAGE loading dye (150 mM Tris-base, 50 mM EDTA, 30% (*v*/*v*), glycerol, 10 % (*w*/*v*) SDS, 0.025 % (*w*/*v*) bromophenol Blue, 50 mM DTT, pH 6.8) and incubated for 5 min at 95 °C before separation of proteins via SDS-PAGE.

For some experiments where IPs were performed after knockdown of Alix, cells were grown in triplicate 6-well plates and treated as described under siRNA transfection. After 24 h of transfection, cells were incubated with fresh media containing antibiotics for a further 24 h for ABCG1-CHOK1 or 48 h for ABCG4-CHOK1. Cell lysates and IP’s were prepared as described above and below.

### 4.5. Cell Lysis and Western Blot Analysis

Cells were washed twice with ice-cold PBS and harvested by adding 150 μL/well of ice-cold 1% IGEPAL with the addition of protease and phosphatase inhibitor cocktails (5 µl/mL). Total cell protein concentrations were measured using a BCA assay and equal amounts of cell protein per lane separated using 10% (*v*/*v*) SDS-PAGE. Proteins were transferred onto nitrocellulose membranes, which were incubated using the following antibody dilutions: anti-Alix (1:2000), anti-NEDD4-1 (1:5000), anti-myc (1:5000), anti-tubulin (1:3000), anti-ABCA1 (1:2500), and anti-ABCG4 (1:5000). HRP-conjugated anti-rabbit or anti-mouse secondary antibodies were used at 1:10,000 dilution. Protein bands were visualized by chemiluminescence and quantified using Image J software (v2.0.0, NIH, Bethesda, MD, USA).

### 4.6. Cholesterol Loading

ABCG1-CHOK1 cells were seeded and transfected with control or Alix siRNA as described above. After 48 h of transfection, the cells were washed twice with PBS and incubated in serum-free medium containing 0.1 % (*v*/*v*) BSA alone or with the addition of filtered solution of cholesterol/ methyl-b-cyclodextrin (Chol/CD complex) at 20 µg/mL for 6 h as described in Luu et al. [29]. The cells were then lysed, and proteins were separated via SDS-PAGE as described above.

### 4.7. Statistical Analysis

Data are expressed as means ± Standard Error of the Mean (SEM). Significance was determined using Student’s *t*-test using Prism software version 7 (GraphPad Software, La Jolla, CA, USA), with *p* < 0.05 considered as significant.
